# The role of oligosaccharides and polysaccharides of xylan and mannan in gut health of monogastric animals

**DOI:** 10.1017/jns.2020.14

**Published:** 2020-06-15

**Authors:** Utsav P. Tiwari, Stephen A. Fleming, Muhammed S. Abdul Rasheed, R. Jha, Ryan N. Dilger

**Affiliations:** 1 Department of Animal Sciences, University of Illinois, Urbana, IL, USA; 2 Department of Human Nutrition, Food and Animal Sciences, University of Hawaii at Manoa, Honolulu, HI, USA

**Keywords:** Arabinoxylan, Immune modulation, Mannan, Oligosaccharides, Polysaccharides, AX, arabinoxylan, A:X, arabinose:xylose, CLTD, carbohydrate recognition domain, DC, dendritic cells, GGM, galactoglucomannan, GH, glycosidic hydrolase, GI, gastrointestinal, MBL, mannose-binding lectin, MOS, mannan oligosaccharide, MR, mannose receptor, PAMP, pathogen-associated molecular pattern, TLR, toll-like receptor, XOS, xylo-oligosaccharide

## Abstract

Apart from its role as a digestive and absorptive organ, the gastrointestinal (GI) tract is a vital immune organ that encompasses roughly 70 % of the total immune cells of the body. As such, the physical, chemical and nutrient composition of the diet influences overall GI function, effectively as an immune organ. With the improvement in feed technology, agro-industrial co-products that are high in fibre have been widely used as a feed ingredient in the diets of pigs and poultry. Arabinoxylan (AX) and mannan are the most abundant hemicellulosic polysaccharides present in cereal grain and co-product ingredients used in the livestock industry. When monogastric animals consume diets containing high amounts of AX and mannans, stimulation of GI immune cells may occur. This involves the activation of several cellular and molecular pathways of the immune system and requires a considerable amount of energy and nutrients to be expended by the animal, which may ultimately influence overall health and growth performance of animals. Therefore, a better understanding of the role of AX and mannan in immune modulation will be helpful in modulating untoward GI immune responses, thereby minimising nutrient and energy expenditure toward this effort. This review will summarise pertinent research on the role of oligosaccharides and polysaccharides containing AX and mannans in immune modulation in order to preserve gut integrity.

## Introduction

Market availability and fluctuation in the cost of conventional feed ingredients are pushing producers to increase inclusion of low-cost, fibre-rich co-products in the diets of monogastric animals. One major issue of this approach is that digestibility of high-fibre ingredients varies widely, with α-linked starch being digestible through hydrolytic–enzymatic action, but β-linked dietary fibres only being digested by exogenously supplied enzymes or through microbial fermentation. Dietary fibre has also been found to contribute to energy utilisation, though this fraction has been long ignored as an insignificant source of usable energy in the diets of monogastric animals. Energy derived from absorption of hexose sugars, like glucose, galactose and mannose, within the small intestine is higher when compared with pentose sugars like arabinose and xylose^(1)^. Nevertheless, monogastric nutritionists are recognising that dietary fibre containing various amounts of a diversity of polysaccharides can elicit immunomodulatory effects within the gastrointestinal (GI) tract. Components of the immune system that have the ability to directly detect polysaccharides include complement proteins, monocytes, macrophages, dendritic cells (DC), neutrophils, lymphocytes and others. Binding of polysaccharides to the immune cell surface receptors triggers several cellular and molecular pathways leading to immune activation, both locally and, in some cases, systemically. Polysaccharide-induced immune stimulation subsequently leads to increased infiltration and proliferation of neutrophils and leucocytes, release of proinflammatory mediators (for example, cytokines, chemokines, vasoactive amines and eicosanoids), elicits formation of reactive oxygen and nitrogen species, and induces a robust synthesis of acute-phase proteins. The entire process of this immune stimulation utilises considerable amounts of nutrients (especially amino acids) and energy^(2)^, which otherwise would have been used for animal growth and productivity.

Arabinoxylan (AX) and mannan are, respectively, the first and second most abundant hemicellulosic polysaccharides present in most cereals and agro-industrial co-products that are used in diet formulations for pigs and poultry^(3)^. Polysaccharides from AX and mannan are considered anti-nutritional due to their negative impact on nutrient utilisation. However, the oligomers from AX and mannan have more functional value in improving the gut health of monogastric animals^(4,5)^. Supplementation of oligosaccharides, or reduction of polysaccharides to oligosaccharides, is one strategy that is helpful in reducing immune stimulation and alleviate untoward effects on gut health caused by polymeric forms within the dietary fibre source^(1)^. Moreover, the nutrient-encapsulating effects of polymers are much lower when they are reduced to shorter-length oligomers^(2)^. Thus, to address these concerns and have a better understanding of the immunomodulatory potential of oligosaccharides and polysaccharides containing AX and mannan, the present review will discuss nutritional concepts and strategies involving AX and mannan when fed to monogastric animals, with specific emphasis on their role in modulating gut health. Given the complex structure of AX and mannan, the present review will discuss the integral relationship between structural heterogeneity of AX and mannan and potential for immunomodulatory effects.

## Gastrointestinal tract: the largest organ of the immune system

The term ‘gut health’ has a very complex and comprehensive definition which includes five distinct domains: (1) a balanced diet that furnishes all required nutrients; (2) a healthy mucosal layer that maintains gut integrity; (3) effective immune responses; (4) neuroendocrine and motor functions of the gut; and (5) a stable microbial community that maintains a balanced, healthy gut environment^(6–8)^.

The GI tract of pigs and poultry represents the largest mass of immune tissue in the body, consisting of more than 70 % of total immune cells^(7)^. Structural and immunological barriers of the GI tract protect the gut from antigens and toxins derived from food. The physical barrier includes epithelial cells, mucus and intercellular tight junctions, whereas the immunological and chemical barrier includes gastric acid, digestive secretions (for example, proteolytic enzymes, lysozymes and antibacterial proteins) and various cytokines and chemokines that regulate chemotaxis of immune cells. Tight-junction proteins regulate intestinal permeability as they act to create a seal between two adjoining enterocytes. Disruption of barrier function, therefore, leads to a condition known as leaky gut syndrome or hyperpermeability^(9)^. Mucosal immunoregulation in the gut is predominantly maintained by single-layered epithelial cells because it acts as the barrier between luminal contents (containing antigens and toxins) and the lymphocyte-rich lamina propria^(10)^. Epithelial cells contain pattern recognition receptors (for example, toll-like receptors; TLR) that recognise antigens and bacterial lipopolysaccharides, which may ultimately cause production of various cytokines and chemokines by local intestinal immune cells in a defensive reaction. When gut health is impaired by the presence of polysaccharides or other immunostimulatory luminal components, it elicits negative effects on feed efficiency and growth performance, ultimately causing economic losses^(8)^. As such, it is imperative to elucidate how dietary components relate to stimulation of immune response within the gut in order to develop nutritional strategies to mitigate such health challenges.

## Xylans and xylanases

**Fig. 1.**
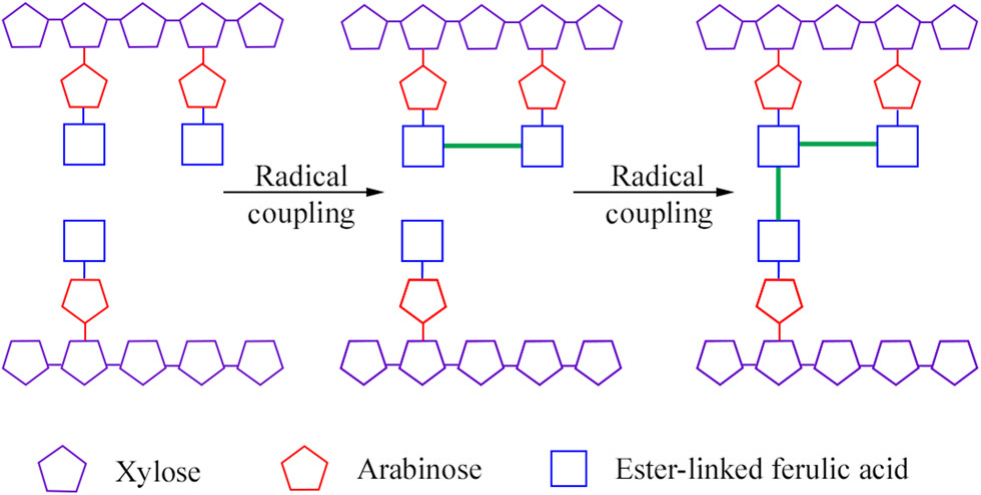
Schematic illustration of coupling (hypothetical) two arabinoxylan chains via ferulic acid (forming dimer and trimer).

Arabinose and xylose occur in the form of AX in feed ingredients. The β(1–4)-linked xylan forms the linear backbone of AX and arabinose gets substituted on the C(O)-2 and C(O)-3 positions, with substitution of hydrocyannamic acid derivatives on the C(O)-5 position^(11)^ (Fig. 1). Arabinose substitution on the xylan backbone (i.e. arabinose:xylose (A:X) ratio) is used to characterise the structure of AX. For example, the A:X ratio in maize is 0⋅74^(12)^, in maize DDGS (distiller's dried grain with solubles) it is 0⋅77^(13)^, in wheat it is 0⋅53^(14)^, in wheat DDGS it is 0⋅66^(15)^ and in macadamia nut cake it is 0⋅88^(16)^. Due to their structural differences, a larger percentage of AX in some ingredients like oats is insoluble, whereas it is soluble in others like rye^(17)^. The molecular weight and A:X ratio of insoluble AX are generally higher than that of soluble AX^(18)^.

**Fig. 2.**
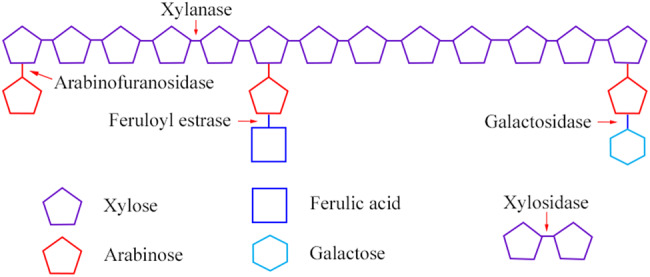
Enzymic attack on arabinoxylan structure.

Xylanases are enzymes that cleave the glycosidic linkage between xylose residues in the xylan backbone. Xylanases are categorised into six different glycosidic hydrolase (GH) families: GH5, GH7, GH8, GH10, GH11 and GH43. Each of the GH families have different substrate specificities. For example, GH10 xylanases cleave xylan molecules with higher degrees of substitution whereas GH11 xylanases cleave unsubstituted xylan. However, GH11 xylanases only cleave those xylan molecules that possess three consecutive xylose without any substitution^(19)^. α-l-Arabinofuranosidase degrades the terminal non-reducing α-arabinofuranose from the AX substrate. The complete degradation of AX requires a combination of enzymes^(19)^ (Fig. 2).

The overall objective of enzyme supplementation via feed is to minimise poor gut health induced by AX and to liberate other nutrients that are entrapped in the fibre, protein and starch matrix. In addition, the liberated shorter fragments of xylo-oligosaccharides (XOS) or arabinoxylo-oligosaccharides may act as prebiotic substrates for microbial fermentation in the large intestine^(20,21)^. The purpose of enzyme supplementation in the feed is not to degrade AX to individual xylose and arabinose as they provide only little energy for the animal. Moreover, most of these monomers will be excreted in the urine^(1)^.

## Mannans and mannanases

Glucomannan, galactomannan, galactoglucomannan (GGM) and linear mannans are the four subfamilies of the mannan family^(22)^. The backbone of all mannan polysaccharides contains β(1–4)-linked mannose or a combination of mannose and glucose residues, which can be substituted with α(1–6)-linked galactose^(23)^. Linear mannan is a homo-polysaccharide containing less than 5 % of galactose substitution^(24)^. Galactomannans consists of β(1–4)-linked mannose units with a single α(1–6)-linked galactose attached to the chain; this form is commonly found within the endosperm of legumes. Glucomannan consists of randomly arranged mannan and glucose residues both in the backbone as well as side chains in the ratio of 3:1 with a degree of polymerisation higher than 200^(23)^. GGM consists of α(1–6)-linked galactose residues attached to glucose or mannose residues as a terminal branch. Partial substitution of O-acetyl groups occurs in mannosyl residues of GGM. In general, GGM consists of galactose on its side chain, which forms strong hydrogen bonds and prevents other macromolecules from aligning to each other, increasing the solubility of GGM^(23)^.

**Fig. 3.**
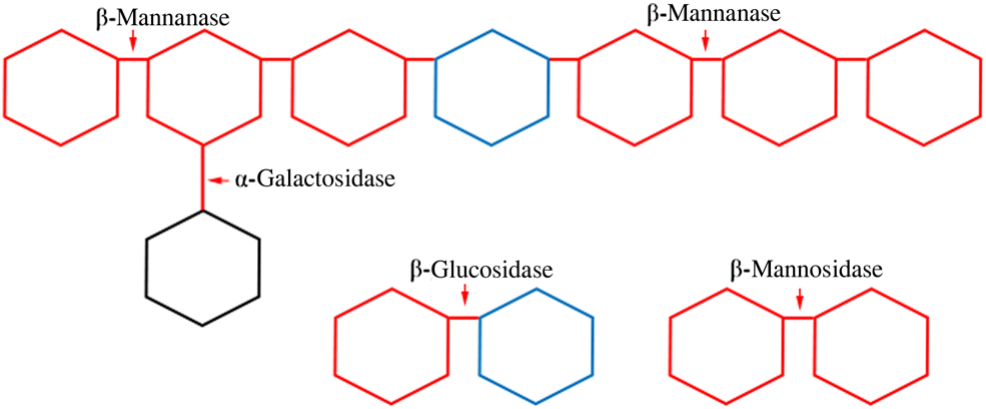
Enzymic attack on galactoglucomannan.

There are mainly three mannan-degrading enzymes: β-mannanases, β-mannosidases and β-glucosidases^(23)^ (Fig. 3). Additional enzymes (α-galactosidases and acetyl mannan esterases) are required to remove side groups from mannans^(25)^. Each of the enzymes act on specific sites and have their own mode of action. β-Mannanases are endohydrolases and they release β-1,4-manno-oligosaccharides^(23,26)^. The β-mannosidases are exo-acting hydrolases, and they release mannose from oligosaccharides. The β-glucosidases remove 1,4-glucopyranose units that are derived from the degradation of glucomannan and GGM. The α-galactosidases help to remove side groups (i.e. α,1–6-linked d-galactopyranosyl units) from the mannan backbone and acetyl groups in the GGM are removed by acetyl mannan esterases.

Digestion and solubility of mannans are affected by the α(1–6) side chain on the β(1–4) mannan backbone. Most of the feedstuffs used in monogastric nutrition are rich in linear mannans or galactomannans, hence β-mannanase and α-galactosidase are the most commonly used mannan-cleaving enzymes. Activity of β-mannanase on a mannotriose substrate was found to occur at a much lower rate^(23)^ compared with mannotetraose and mannopentaose substrates, signifying greater efficacy of β-mannanase if the mannan backbone consists of more than three contiguous mannose residues.

## Dietary polysaccharides and immune modulation

Many researchers have investigated the immunomodulatory effect of dietary polysaccharides, though most of them have focused on bacterial lipopolysaccharides which contain lipid A. However, the polysaccharides present in feed ingredients provided to monogastric animals are of plant origin that do not contain lipid A but still possess immunomodulatory properties^(27)^. Immune activation causes the shift of energy utilisation from growth to support immune defence pathways, ultimately inhibiting the productive performance of the animal^(2)^. However, the exact mechanisms by which dietary fibrous polysaccharides influence the immune response and inflammation are not fully understood. Three possible pathways highlight the immunomodulatory potential of dietary fibrous polysaccharides fed to monogastric animals: (1) direct interaction with epithelial cells or leucocytes; (2) uptake by M-cells in the Peyer's patches; and (3) uptake by macrophages and DC. Polysaccharide uptake by M-cells results in transcytosis to immune cells and subsequent cytokine production, whereas uptake by macrophages results in transportation of the polysaccharides to immune tissues including lymph nodes, spleen and bone marrow. Several studies conducted on monoculture cell lines with polysaccharides report that the cell wall polysaccharides may directly stimulate epithelial cells and monocytes, subsequently causing immunological outcomes^(19,28,29)^.

Larger polysaccharides are recognised by pattern recognition receptors (PRR) found on immune cells that survey and guard peripheral tissues; these cells stimulate the production of cytokines and proliferation of lymphocytes. The PRR are sensors of the immune system and are present on and in immune and intestinal epithelial cells. The PRR involved in the recognition of NSP are TLR and C-type lectin receptors^(4)^. Currently, ten TLR have been identified in monogastric animals and each accept distinct pathogen-associated molecular patterns (PAMP) derived from various pathogens. The PAMP represent unique molecular signatures that are only present on microbes and not on host cells. Certain TLR (1, 2, 4 and 6) recognise lipoproteins (for example, triacyl lipopeptides), whereas other TLR (3, 7, 8 and 9) recognise nucleic acids of microbial origin (either single- or double-stranded RNA)^(20)^. Besides bacteria, viruses, lipids, protein and nucleic acids, TLR are also able to recognise carbohydrate moieties. For example, inulin-type fructans modulate TLR2 to enhance barrier function and prevent entry of pathogens, yet C-type lectin receptors exclusively bind to NSP (for example, dectin-1 recognising β-glucan) and tend to cause immunostimulation^(30,31)^.

### Arabinoxylan and immune modulation

Receptors involved in the recognition of AX have not been identified, though it has been suggested that AX may act like PAMP^(4,19)^. The structure and molecular weight of AX derived from maize are similar to those of bacterial lipoproteins^(32)^. Both AX and bacterial lipoproteins have C-3-branched polysaccharides and an outer core of hexoses such as glucose and galactose^(30)^, which means that both are recognised by TLR4. Additionally, AX has been proven to elicit an immune response in chickens experiencing an *Eimeria* parasitic infection^(33)^. The AX derived from wheat bran was found to have higher immunostimulatory activity than AX derived from maize husk^(34)^, supporting the notion that AX structure is related to the ability for recognition by host immune cells. Maize has more heavily substituted arabinose units as compared with wheat^(13,15)^, and reflected in a higher A:X ratio in maize (0⋅83) *v.* wheat (0⋅56), suggesting arabinose enrichment may be partly associated with higher immunostimulatory activity.

### Mannan and immune modulation

Two mechanisms have been proposed by which β-mannan modulates immune responses through direct interaction: (1) via the cell surface mannose receptor (MR) that can identify both host glycoproteins and microbial glycans^(35)^; or (2) via mannose-binding lectins (MBL) that prompt inflammatory response through cytokine expression^(36)^. Because the location of host receptors may relate to immunostimulatory action, it is important to note that the MR is membrane bound whereas MBL are secreted by the liver^(1,2)^.

The MR is a member of the MR family which consists of four members: (1) MR proper; (2) M-type phospholipase A2 receptor (A2); (3) Endo 180 (endocytic glycoprotein); and (4) DEC 205 (cell surface protein primarily expressed by DC). The general structure of all the members of the MR family is similar in that they consist of an extracellular region containing a cysteine-rich domain, a repeating fibronectin type-2 domain, multiple C-type lectin-like carbohydrate recognition domains (CLTD), a transmembrane domain and a cytoplasmic tail^(37,38)^. The MR, Endo 180 and phospholipase A2 contain eight carbohydrate recognition domains whereas DEC 205 contains ten. Out of the eight CLTD in MR and Endo 180, only the 4th and 5th CLTD in MR and the 2nd CLTD in Endo 180 can directly bind mannose in a Ca-dependent manner^(35)^. The MR is an endocytic receptor. Binding of mannan to MR induces endocytosis followed by its delivery through the endosomal pathway^(35)^ (i.e. uptake of mannan and presentation to T cells), which requires coordination with other PRR like TLR4^(35,37)^.

MBL is a soluble C-type lectin receptor synthesised in the liver as part of the host acute-phase response and binds to neutral carbohydrates (for example, l-fucose, d-mannose and *N*-acetylglucosamine) on the microbial surface^(39)^. Unlike other acute-phase proteins (for example, haptoglobulin) that increase 10- to 1000-fold in the serum, MBL fluctuates only 2- to 3-fold during an inflammatory reaction^(40)^. The repeating linked array of mannans located within plant-based β-mannan polymers provides potential targets for MBL binding^(40,41)^. As such, MBL activates the lectin pathway of the complement system by binding to specific carbohydrates with the help of the mannan-binding, lectin-associated serine protease. The complement system is activated by three pathways (classical, properdin and lectin pathways) and is activated by microbes and MBL, which involve similar pathways^(20)^.

Mannans do not get hydrolysed by endogenous host enzymes in monogastric species, therefore they are available for binding to the carbohydrate recognition domains of innate immune cells, including macrophages, to elicit an immune response^(33)^. In *in vitro* studies mannan was found to stimulate macrophages and DC, causing increased pro-inflammatory cytokine production^(42,43)^. DC activation plays a key role in the induction of immune responses, but the efficacy of the DC-induced response depends upon the developmental state of the host DC^(43)^. Galactomannan induces phenotypic maturation of DC with increased expression of pro-inflammatory cytokines at both the transcriptional and protein levels^(43)^. Activation of DC leads to increased antigen presentation, increased cytokine secretion and ultimately an optimal T-lymphocyte activation^(44)^. Secretion of IL-6 and TNF-α induced by galactomannan establishes an inflammatory environment that helps to initiate and maintain DC activation. Galactomannan interacts with DC through mannan receptors, probably due to the highly branched mannose structure. Additionally, TLR4 present on the surface of DC also has a high affinity for mannan. Galactomannan has an outer core of hexose moieties (especially glucose and galactose); hence they are able to bind TLR4 and induce an immune response. Moreover, the β-mannan in *Aloe vera* and soyabean have similar chemical structures and are found to activate macrophages and induce NO synthesis^(5)^. NO acts as cytostatic factor that inhibits proliferation of viruses and a wide range of invading pathogens^(33)^. Exogenous mannanase supplementation has been found to decrease haptoglobin production (i.e. down-regulate the acute-phase protein response), thereby suggesting decreased immune stimulation may occur with lower-molecular-weight mannans.

## Role of functional groups attached to polysaccharides

All of the NSP present in the cell wall of cereal grains are composed of monosaccharides and each monosaccharide can exist in two ring forms, namely pyranose and furanose linked by glycosidic bonds in either the α or β orientation^(12)^. As a result, these polysaccharides can form different types of three-dimensional shapes, which permits a vast array of functional groups. The presence of functional groups like acetate and/or sulfate on the cell surface is known to affect the charge and solubility of polysaccharides, with sulfate groups providing negative charges and acetyl groups forming hydrophobic pockets^(4)^.

In addition to influencing chemical properties of the substrates *per se*, acetylation, sulfation and degree of branching of fibrous polysaccharides also influence gut condition, microbial ecology and immunostimulatory potential in the host consuming those polysaccharides via the diet. A higher degree of acetylation has been found to lower the immunostimulatory effect of mannan^(4)^. However, partially O-acetylated AX induces proliferation of thymocytes, indicating increased immunostimulatory activity^(45)^. Sulfation of galactans has been reported to have immunostimulating activity as sulfated and carboxymethylated functional groups of β-glucan have been proven to improve solubility by impacting inter- and intra-molecular hydrogen bonding and strengthening electrostatic repulsion ability^(4)^.

Branching, or the degree of substitution on the backbone of fibrous polysaccharides, influences the way a dietary substrate interacts with the GI environment and ultimately affects the immune response. A higher A:X ratio leads to increased cross-linking, making it difficult for the enzymes to act upon the substrate^(13,46)^. Cross-linking is also associated with the presence of hydroxycinnamic acid derivatives, which are most commonly found in cereals as ferulic, coumaric and sinapic acids^(11)^. Ferulic acid is the predominant form in most cereals, with the concentration of ferulic acid being highest in maize, moderate in wheat and lower in oats^(17)^. AX in the aleurone layer is mostly insoluble due to its high ferulic acid content and heavy esterification of AX of the aleurone layer^(15)^. This means that more cross-linked complex polysaccharides are present in maize and its co-products than wheat and oats. Additionally, more than 90 % of ferulic acid occurs in the bound form whereas the free and soluble forms are found at lower concentrations. Thus, occurrence of ferulic acid in the insoluble fraction of fibre is 100-fold higher than in the soluble fraction^(17)^. As such, ferulic acid is thought to form a bridge between polysaccharide chains, and thereby influence the physiochemical properties of fibre and effectively lower the efficiency of exogenous enzymes consumed by the host.

## The role of solubility, viscosity and molecular weight of polysaccharides

The solubility of fibres in the gut is dependent on the type of ingredients used during the feed formulation process. Soluble fibre is better degraded by enzymes and more easily fermented by microbes in the large intestine of pigs^(13)^, which may relate to the concentrations of diferulates present in the respective fibrous fractions^(47)^. Moreover, the concentration of diferulates present in the insoluble fraction of wheat is 5–7 times higher than that present in the soluble fraction of wheat^(11)^. Higher amounts of insoluble fibre are concurrent with higher levels of diferulates and increased cross-linking^(11,13)^. Finally, solubilised AX has a larger fraction of reducing ends that makes AX more susceptible to Maillard reactions^(15)^.

Fibre viscosity is another property that is positively associated with the molecular weight of polysaccharides present in feed ingredients. For example, the molecular weight of β-glucan is higher than that of AX^(48)^ and β-glucan is far more viscous than AX when in solution^(18)^. However, β-glucan is also more sensitive to reduction of molecular weight during passage through the small intestine^(12)^, while AX is more resistant to degradation under the same conditions, which may help to explain the greater extent to which AX negatively influences nutrient digestibility *v.* β-glucan.

Presence of higher concentrations of soluble fibre is also associated with increasing the viscosity of intestinal content, as soluble fibres absorb water and increase their bulk. Inclusion of high amounts of soluble AX and mannan (>0⋅4 %) in a poultry diet would increase digesta viscosity, causing an increase in epithelial cell proliferation inhibiting nutrient digestibility^(5)^. Insoluble linear mannans with lower galactose substitution (for example, palm kernel meal and soyabean mannans) do not form any viscous solution in the GI tract of monogastric animals, whereas mannans with a high degree of galactose substitution (for example, copra meal or guar meal) cause a stark increase in digesta viscosity^(5,21)^. An increase in viscosity of luminal contents is associated with an increase in the rate of villus cell loss, thereby leading to villus atrophy^(49)^, slowed gastric emptying and a reduction in the contact of nutrients with enterocytes and digestive enzymes^(13)^. Collectively, these alterations elicit a negative impact on overall nutrient availability of the monogastric diet. Since soyabeans are used as a major source of protein in most of the feed fed to monogastric animals, the presence of mannan can be presumed in most of the feeds. However, soya mannan is mainly linear mannan (containing less than 5 % galactose); they do not play a significant role in increasing intestinal viscosity^(5)^.

Long villi and shallow crypts help to increase absorption of nutrients by providing a larger surface area and lower proliferation and renewal rates of enterocytes constituting villus structures results in more efficient enterocyte maturation and enzyme production^(21)^. Crypt depth is also associated with cellular replacement rates as increased turnover requires more energy and amino acids, ultimately impacting growth of monogastric animals by shifting resources away from productive processes involving saleable products (i.e. meat and eggs). In monogastric animals, approximately 20 % of all the dietary energy is used by the GI tract to maintain structures supporting digestive and absorptive processes^(7)^. Supplementation of moderate amounts of guar meal (5–8 %) causes a marked increase in digesta viscosity in broilers^(5)^, which has been proved to increase proliferation of *Escherichia coli* and *Clostridium* within the GI tract^(28)^. Laying hens have increased digestive capacity compared with broilers and evidence suggests that the untoward effects of dietary fibre-induced viscosity are lower in layers compared with broilers fed similar concentrations of NSP^(5)^. Dietary supplementation of xylan- and mannan-degrading enzymes will serve to break down polymeric fibre structures to shorter oligomeric forms, and thereby reduce viscosity of intestinal contents, enhance gut health and improve performance of monogastric species.

## Oligosaccharides of xylan and mannan

Oligosaccharides are low-molecular-weight carbohydrates and the degree of polymerisation can vary from 2 to 10; however, molecules with a degree of polymerisation of less than 20 are sometimes considered as oligosaccharides due to their prebiotic potential. Prebiotics are non-digestible food ingredients that beneficially affect the host by selectively stimulating the growth and/or activity of one or a limited number of bacterial species already resident in the colon^(50)^. The XOS and mannan oligosaccharides (MOS) are newly developed functional oligosaccharides produced from xylan and mannan, respectively, by enzymic hydrolysis that have beneficial immunological and health effects. Depolymerisation of XOS and MOS into oligosaccharide configurations by use of enzymes *in situ* (i.e. prior to feeding to monogastric animals) is neither instantaneous nor as efficient as supplementing dietary XOS and MOS directly^(5,51)^. Supplementing XOS and MOS has beneficial effects comparable with in-feed antibiotics in reducing gut inflammation^(19,21)^. These oligosaccharides are intermediate between simple sugars and polysaccharides and behave both as dietary fibres and prebiotics.

XOS and MOS are linked together by the β-glycosidic bond of the linear polyxylose or polymannose chains, respectively^(52)^. Several studies have found XOS to selectively increase the proliferation of bifidobacteria to a larger extent than fructo-oligosaccharide (FOS)^(53)^ or any other oligosaccharides^(54)^. This ‘bifidogenic effect’ of XOS has been shown in both *in vitro*^(55)^ and *in vivo* studies^(56)^. The XOS and MOS with monomer units of 5 or fewer are getting more attention for their potent prebiotic and butyrogenic effect.

XOS are thermostable during pasteurisation and can be autoclaved at a lower pH as compared with fructo-oligosaccharide (FOS), which is more vulnerable to decomposition at lower pH and higher temperature^(45)^. Manno-oligosaccharides are also heat resistant as they can tolerate a higher temperature of up to 120°C for up to 20 min^(21)^. Hence, both XOS and MOS should theoretically be resistant to destructive processes involved in various feed milling operations, including pelleting and some forms of extrusion. As such, XOS and MOS not only resist salivary hydrolysis or hydrolysis by gastric and pancreatic secretions, they are also not absorbed within the small intestine^(57)^, hence providing a substrate for microbial fermentation in the colon of pigs and caecum of poultry^(58)^. The difference between oligosaccharide and polysaccharides of xylan and mannan is that XOS and MOS cannot form cross-linkages and are not able to stimulate immune receptors^(8)^. Thus, XOS and MOS may help to reduce immune stimulation in monogastric animals and all the associated metabolic costs involved to maintain the immune response.

## Bifidogenic effect of oligosaccharides

Host and gut microbes interact with each other to influence various physiological functions of the host. Functional oligosaccharides from xylan and mannan have been used in many studies to manipulate the gut microbial ecology of monogastric animals. Numerous beneficial effects on host health have been reported by oligosaccharide-mediated changes in gut microbiota including activation of the immune system^(59,60)^, production of antimicrobial factors^(56,61)^, exclusion of specific enteric pathogens and changes in gut histomorphological structure^(62)^. Bacteroidetes and Firmicutes are the polysaccharide-degrading phyla possessing the largest set of glycoside hydrolase-encoding genes^(36,63)^. On the other hand, oligosaccharides are a class of compounds that help the proliferation of beneficial microbes like bifidobacteria and *Lactobacillus* to improve the health of animals. The role of bifidobacteria in the utilisation of oligosaccharides is gaining more attention because of their stringent selectivity of carbohydrate substrates based on the degree of polymerisation. While the majority of other enteric bacteria predominantly ferment monosaccharides, bifidobacteria preferentially utilise disaccharides and other oligomeric forms over monomeric and polymeric forms^(58)^.

Polymeric xylans and mannans are degraded by *Bacteroides* and *Roseburia*, whereas the oligomeric form is only degraded by bifidobacteria and a few species of Firmicutes, including *Lactobacillus brevis*^(63,64)^. No other *Lactobacillus* sp. are capable of degrading oligomeric forms of xylan as a sole carbon source^(65)^. As an exception, *L. brevis* can only degrade unsubstituted XOS^(66)^. Uptake of oligomers of AX is mediated by the ATP-binding cassette transport system, and the only known gut microbes capable of producing this transport system is bifidobacteria^(63)^. Most of the *Bifidobacterium* sp. are shown to degrade AX, but there is variation in their degradation capabilities. *Bifidobacterium adolescentis* and *B. vulgatus* can completely degrade AX^(67)^, because they are capable of degrading both substituted and unsubstituted arabinose^(68)^. While *B. longum* and *B. ovatus* can partially degrade AX, *B. breve* and *B. infantis* cannot degrade XOS at all^(67)^. The xylan-degrading enzymes produced by different *Bifidobacterium* sp. differ in their specificity and efficiency, though they have highly conserved solute-binding proteins that enable the degradation of AX oligomers^(63)^. This may partly explain the variation in degradation of AX polymers exhibited by different *Bifidobacterium* sp.

*Bacteroides* can only degrade those oligomers which are larger than xylo- and manno-pentaose^(64)^. Higher bifidogenic effects may be achieved when feeding XOS with higher arabinose substitution when compared with *Bacteroides*^(19)^. *Bacteroides xylanisolvens* growth was hindered when xylotriose with di-substituted arabinose was used^(69)^, indicating that arabinose-substituted XOS may adversely affect bacterial growth.

*Bacteroides*, *Bacillus* and *Clostridium* are the three bacterial genera which have the best ability to produce mannanase that can degrade mannans^(3)^. MOS is found to increase Bacteroidetes proliferation^(33)^ and many studies have reported *Lactobacillus* as the main genus that is influenced by MOS. However, among all different strains of *Lactobacillus*, *L. salivarius* is most effective against *Salmonella* colonisation and *L. crispatus* against both *Salmonella* and *E. coli*^(33)^. Supplementation of 0⋅2 % dietary MOS to broilers significantly reduced profiles of *E. coli* in the caecum and ileum of birds^(21)^. Bacterial diversity in the GI tract of monogastric animals is high, which assists in enabling various functions, including degradation of NSP that cannot be digested by host enzymes. Thus, XOS and MOS can be used as a dietary intervention to enhance the host's natural defence through modulation of gut microbiota. Supplementation of NSP-degrading enzymes can also have an impact on the microbial ecology of the gut^(70)^ that can reduce the amount of undigested substrates, degrading polymers to oligomers that potentially have functional prebiotic effect.

## Oligosaccharide effects on gut barrier functions

The intestinal lining has a versatile function that helps to regulate nutrient and water absorption, while at the same time acting as a barrier excluding microbes and infectious agents that come into contact with the gut. The harmful effects elicited by pathogenic microbes occur after they attach to enterocytes of the intestinal wall and colonise the environment. However, current evidence suggests that dietary oligosaccharides may assist in clearing pathogenic microbes from the GI tract, partly through the production of mucins by goblet cells embedded among enterocytes^(21)^. Mucin secretion at the brush-border membrane limits the number of bacteria that reach the epithelial layer and influence the metabolic activity of bacteria. Increased mucin production helps to improve gut barrier function as pathogenic microbes cannot penetrate the dense mucous layer^(36)^. XOS have been found to increase goblet cell number and density^(21,71)^, which also increases mucin secretion (i.e. glycoproteins) and protein barrier factors^(72)^, thereby protecting intestinal epithelial cells. Similarly, MOS has anti-pathogenic effects, because of its ability to increase the number of sulfated goblet cells and concomitantly increase mucin secretion. Goblet cells sulfated by the use of MOS have been shown to provide greater protection for the host by enhancing pathogen degradation and reducing pathogenic microbe attachment to colonic epithelial cells^(73)^. Moreover, soluble mannosyl receptors secreted in mucin competitively bind to type-1 fimbriae of pathogenic microbes like *Salmonella*, *E. coli* and other Gram-negative bacteria. Thus, increased mucin production in the gut due to the presence of MOS helps in clearing pathogens by preventing microbial attachment to the intestinal wall^(21)^.

Both XOS and MOS play important roles in maintaining gut integrity by increasing the secretion of IgA. Generally, mucin and secretory IgA are considered a first line of defence against the establishment of pathogenic micro-organisms in the ileum through the prevention of adhesion and subsequent epithelial invasion. Almost 98 % of all IgA-producing cells in the body reside in the small intestine^(74)^. IgA is different from any other immunoglobulin as it removes antigens/pathogens without prompting inflammatory immune responses^(75)^. Thus, increased secretory IgA in the small intestine promotes an efficient prevention of intestinal inflammatory conditions. As such, feeding XOS via wheat bran increased the concentration of secretory IgA, which was shown to protect mucosal epithelia by preventing the attachment of pathogenic microbes^(76)^, strengthening tight junctions between epithelial cells and down-regulating the production of pro-inflammatory cytokines like TNF-α, IL-6 and interferon-γ^(56)^. Among MOS, mannobiose has been found to be the most effective at preventing bacterial infections by increasing IgA production in both pigs^(59)^ and chickens^(77)^. Supplementation of MOS increased the concentration of mucosal IgA and reduced oocyte shedding in the excreta of broilers, presumably via secretory IgA inhibiting oocyte development^(78)^. Additionally, supplementation of mannobiose in cultured chicken macrophage cell lines caused increased elimination of *Salmonella*^(60)^. This increase in phagocytic activity against *Salmonella* might be due to increased production of H_2_O_2_ and NO. Thus, improvement in the intestinal barrier function by the use of XOS and MOS helps prevent pathogen colonisation and enhance overall immunity and intestinal health of monogastric livestock species.

## Conclusion

AX and mannan are the major NSP present in most of the cereals and agro-industrial co-products used in the diet of monogastric animals. The immunomodulatory properties of AX and mannan are associated with their sugar composition, structure, molecular weight, solubility, presence of functional groups and degree of branching. Both AX and mannan have structural similarities with lipoproteins in terms of C3 branching and in the presence of an outer core of hexose sugars, which suggests immune responses caused by dietary AX and mannan may be due to the activation of lipoprotein receptors in the host. Reduction of high-molecular-weight polymers to lower-molecular-weight oligomers increases their solubility in water and thereby elicits gut health benefits. In addition, fermentation of AX and MOS in the distal GI tract aids in maintaining gut health by preventing pathogen attachment. Inclusion of polysaccharide-degrading enzymes or direct supplementation of extracted oligosaccharides derived from AX and mannan may be an effective strategy to mitigate the negative effects of NSP ingestion, thereby improving intestinal health and overall performance of monogastric animals.
